# An Fiber Bragg Grating-Based Monitoring System for Slope Deformation Studies in Geotechnical Centrifuges

**DOI:** 10.3390/s19071591

**Published:** 2019-04-02

**Authors:** Lei Zhang, Bin Shi, Luigi Zeni, Aldo Minardo, Honghu Zhu, Lixiang Jia

**Affiliations:** 1 School of Earth Science and Engineering, Nanjing University, Nanjing 210023, China; zhh@nju.edu.cn; 2 Department of Engineering, University of Campania “Luigi Vanvitelli”, 81031 Aversa, Italy; aldo.minardo@unicampania.it; 3 Suzhou NanZee Sensing Technology Co. Ltd., Suzhou 215123, China; jialx@nzsensing.com

**Keywords:** geotechnical centrifuge, optical fiber sensors, fiber Bragg grating (FBG), monitoring system, slope deformation

## Abstract

Centrifugal model tests, which can reproduce the deformation process of the slope, play a crucial role in investigating the mechanism of slope failure. The FBG-based sensors, with high precision, electromagnetic resistance, light weight and small size, have been introduced into geotechnical centrifuge monitoring. The slope evolution is a complex multi-parameter dynamic process which involves the interaction of displacement, stress and strain. However, current research is mainly focused on one or two monitoring aspects, i.e., strain or displacement monitoring to study some specific questions. To achieve multi-parameter and real-time monitoring, a comprehensive fiber Bragg grating (FBG) monitoring system including miniaturized anchors, earth pressure gauges, inclinometer pipe and retaining wall, has been designed for geotechnical centrifuge tests. Before the centrifugal test, laboratory calibrations of sensors were carried out. The calibration results indicate that the FBG-based sensors can monitor the strain, stress and displacement variation precisely. The multi-parameter information related to slope stability were captured and analyzed in detail. The stress state of the anchors, strain distribution of retaining wall together with the displacement of the inclinometer pipe indicate the progressive evolutionary process of the model slope. The test results also indicate that the critical centrifugal force for the transition of the sliding surface is 45 g, after which, a sliding surface is formed in the soil above the retaining wall. The feasibility and validity of the monitoring system is verified by a comparison between the results of FBG-based sensors and those of a numerical simulation. In summary, the innovative FBG-based monitoring system has provided a feasible multi-parameter monitoring method in geotechnical centrifugal tests so as to facilitate further in-depth analysis.

## 1. Introduction

In geological, geotechnical and environmental engineering, centrifugal tests for small-scale physical slopes can reproduce the deformation process of large-scale slopes [1,2], which is an important method for understanding the behavior of slopes. A reliable centrifugal monitoring system is critical to obtain complete and high-quality model test results. Particle image velocimetry (PIV) [3,4,5], CT scanning [6] and other image-recording technologies [7,8,9] are the commonly used methods to obtain displacement information. However, the deformation of a slope is a complex and dynamic evolution process, while the image-record technology mentioned above can only obtain the surface deformation information, instead of a clear understanding of internal deformation. Therefore, accurate evaluation of slope stability conditions is hard. Measurement of the internal information (i.e., displacement, strain and force) and its variation can be helpful for comprehensive model analysis. Therefore, numerous centrifuge test use in-flight installation of structures to investigate triggered factors, excavation-induced slope instability, and interaction with other structures [10,11,12]. In-flight structures (i.e., anti-slide piles, retaining walls, and anchors) with attached electric resistance strain gauges not only can monitor the health conditions, but also evaluate the stability of a landslide. However, the most commonly used electric resistance strain gauges (BX120-3AA) are 8 mm in length and 5 mm in width, which means that the substrate must be bigger than 5 mm in diameter. Besides, each electric resistance strain gauge has independent transmission wires. When numerous electric resistance strain gauges are used in the test, the massive and complex routing of the transmission wires will change the properties of the substrate and soil, affecting the accuracy of the results. 

In the past decade, the fiber Bragg grating (FBG) sensing technology has developed very rapidly [13]. The fiber optic sensors have unique advantages over traditional transducers (i.e., strain gauges, piezometer, tensiometer,…), including immunity to electromagnetic interference, high precision, excellent long-term monitoring and the ability of multiplexing [14,15]. Therefore, some researchers have begun to introduce this technology into civil and geotechnical engineering. A FBG-based soil anchor was designed, whose strain distribution under loading conditions was measured and analyzed [16]. A slope disaster early warning system was demonstrated, by recording the deformation information of the FBG-based anchor bars in real time [17]. Correia et al. have developed a novel FBG-based effective soil pressure sensor, achieving simultaneous measurement of total soil pressure and pore pressure [18]. A laboratory test with different buried FBG sensors was carried out and the evolutionary process of the model slope was revealed with monitored physical information [19]. Hong et al. has introduced the recent development and application of FBG-based sensors for health monitoring, including soil nail systems, inclinometers, piles, etc. [20]. The various applications of FBG-based sensors have achieved good results, however, most of them are not suitable for small-scale centrifugal tests, therefore, it is necessary to develop sensors especially suitable for centrifuge tests. Zhang et al. [21] conducted a FBG-based centrifuge model test to investigate the internal deformation of soil and the failure of a slope. However, the bare FBG arrays easily break when the slope faces a large deformation. The strain variation during the pressing-in of piles with FBGs attached was monitored and the relationship between the lateral friction resistance and the S/d ratio was revealed in a centrifugal model test [22]. The centrifugal test carried out by Wang et al. was to study a specific question, the performance of piles used to reinforce loess foundations. An FBG-based system was developed and the failure process was investigate, by monitoring the surface displacement and strain changes in the anchors and anti-sliding piles [23], but the surface displacement is merely the sum of the longitudinal displacement at this point, which can’t reflect the internal displacement distribution. Besides, only two FBGs were mounted on the anchor and anti-sliding pile, which can only provide limited data, with the risk of missing critical points. Therefore, it provided only some qualitative cognition. The research mentioned above provides insight into the deformation mechanism and evolution process of centrifuge model slope, thanks to novel FBG-based sensors. While the deformation of slope is the result of interactions of a multi-parameter (displacement, strain, stress) dynamic process [24], current centrifugal studies are mainly focused on one or two aspects, i.e., strain or displacement monitoring, which may compromise an overall understanding of the slope failure process. For those reasons, a comprehensive multi-parameter FBG-based monitoring system is proposed in this paper, which can achieve multi-parameter monitoring synchronously. Laboratory calibration tests have been carried out using the FBG monitoring system in the centrifugal model slope. The centrifugal force has been varied from 5 g to 60 g, and the results of FBG-based sensors have been obtained and analyzed. The results confirm that the FBG monitoring system achieves multi-parameter measurement at different scales with high efficiency.

## 2. FBG-Based Sensors: Principle and Design

### 2.1. Working Principle

**Figure 1:**
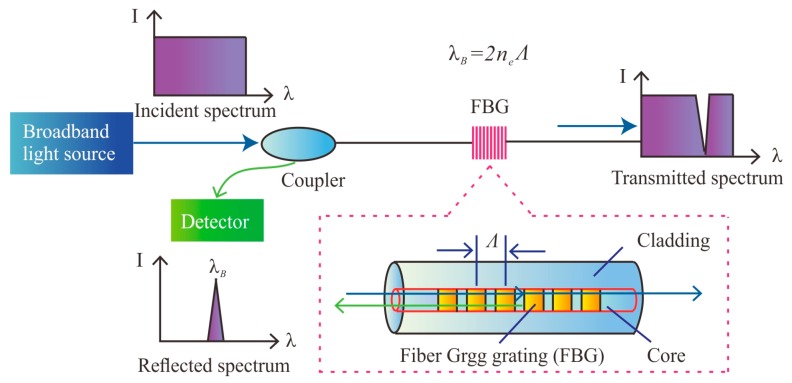
Measuring principles of FBGs.

Figure 1 shows the working principle of an FBG. The fiber Bragg grating is fabricated into the core of a single-mode fiber [25]. When a broadband light source is injected into the fiber, the FBG reflects a narrowband portion of the incident light around a certain wavelength, which can be expressed as [26]:

(1) $$\lambda_{B}=2n_{e}\Lambda$$

where *λ_B_* is the so-called Bragg wavelength; *n_e_* is the effective refractive index; and *Λ* is the grating pitch. The Bragg wavelength is sensitive to the axial strain and the temperature. The linear relationship between the Bragg wavelength (λ*_B_*) and the applied strain (Δ*ε*) and temperature (Δ*T*) is expressed in Equation (2):

(2) $$\frac{\Delta \lambda_{B}}{\lambda_{B}}=(\alpha_{f}+\xi )\Delta T+\left(1\;-P_{e}\right)\Delta \varepsilon$$

where Δ*λ_B_* is the shift of the Bragg wavelength, *P_e_* is the photo-elastic coefficient, α*_f_* is the thermal expansion coefficient, *ξ* is the thermal-optic coefficient.

The axial strain of a structure can be measured by the FBGs attached to it. After eliminating the temperature influence, the axial force of the structure satisfies the following relationship [27]:

(3) $$\begin{array}{c} \sigma \left(z\right)=\varepsilon (z)\cdot E_{C}\\ Q\left(z\right)=\sigma (z)\cdot A \end{array}$$

where *E_c_* is the elastic modulus of the anchor, *A* is the cross-sectional area of the anchor, *σ*(*z*) is the axial stress of anchor, *ε*(*z*) is the strain measured by the FBGs glued on the anchor, and *Q*(*z*) is the axial force of the anchor. 

**Figure 2:**
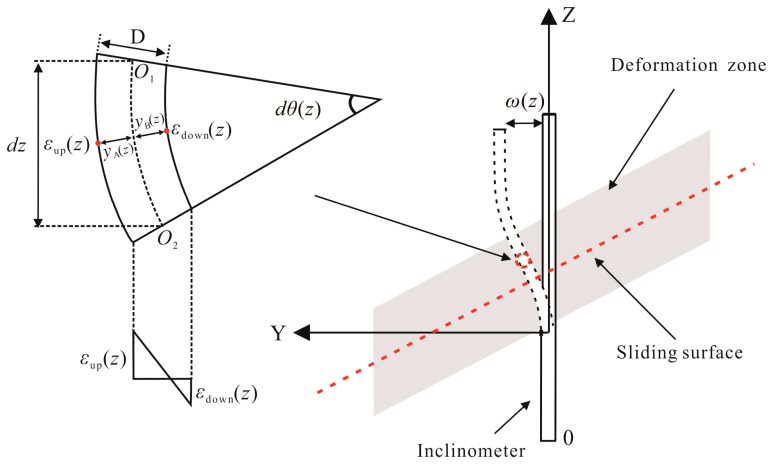
Principle of FBG inclinometer displacement calculation.

The schematic diagram of an FBG-based inclinometer is shown in Figure 2. Based on the classic Euler beam theory, the lateral displacement of the inclinometer can be calculated from the FBG strain data. As shown in Figure 2, the relationship between the strain induced by bending and the radius of curvature at any point of the inclinometer along the main slip direction can be expressed as [28]:

(4) $$\begin{array}{c} \frac{1}{\rho (z)}=-\frac{\varepsilon_{m}(z)}{y(z)}\\ \frac{1}{\rho (z)}=\frac{d^{2}\omega (z)}{dz^{2}} \end{array}$$

where *dz* is the length of segment o_1_o_2_ along the neutral axis, *ε_m_* (*z*)is the strain of the inclinometer and *ρ*(*z*) is the radius of curvature of segment o_1_o_2_, *y*(*z*) is the distance between the strain test point and the curve neutral plane of the inclinometer. The deflection of the inclinometer, *ω*(*z*), can be obtained through Equation (4):

(5) $$\omega (z)=\int_{0}^{z}\int_{0}^{z}\frac{\varepsilon_{m}(z)}{y(z)}dzdz+Az+B$$

As the bottom of the inclinometer is fixed, it can be simplified as a cantilever beam structure. According to the boundary condition, A = B = 0, the derived Equation (6) is herein applied to calculate the deflection of the inclinometer:

(6) $$\omega (z)=\int_{0}^{z}\int_{0}^{z}-\frac{\varepsilon_{u}(z)-\varepsilon_{d}(z)}{D}dzdz$$

### 2.2. Sensor Design

As the geotechnical centrifuge test model is generally 0.03~2.25 m^3^, the size of the FBG sensors used should be small. In view of this, miniaturized FBG-based sensors were designed, including anchors, inclinometer pipe, retaining wall and earth pressure gauges. 

#### 2.2.1. Anchors and Inclinometers

**Figure 3:**
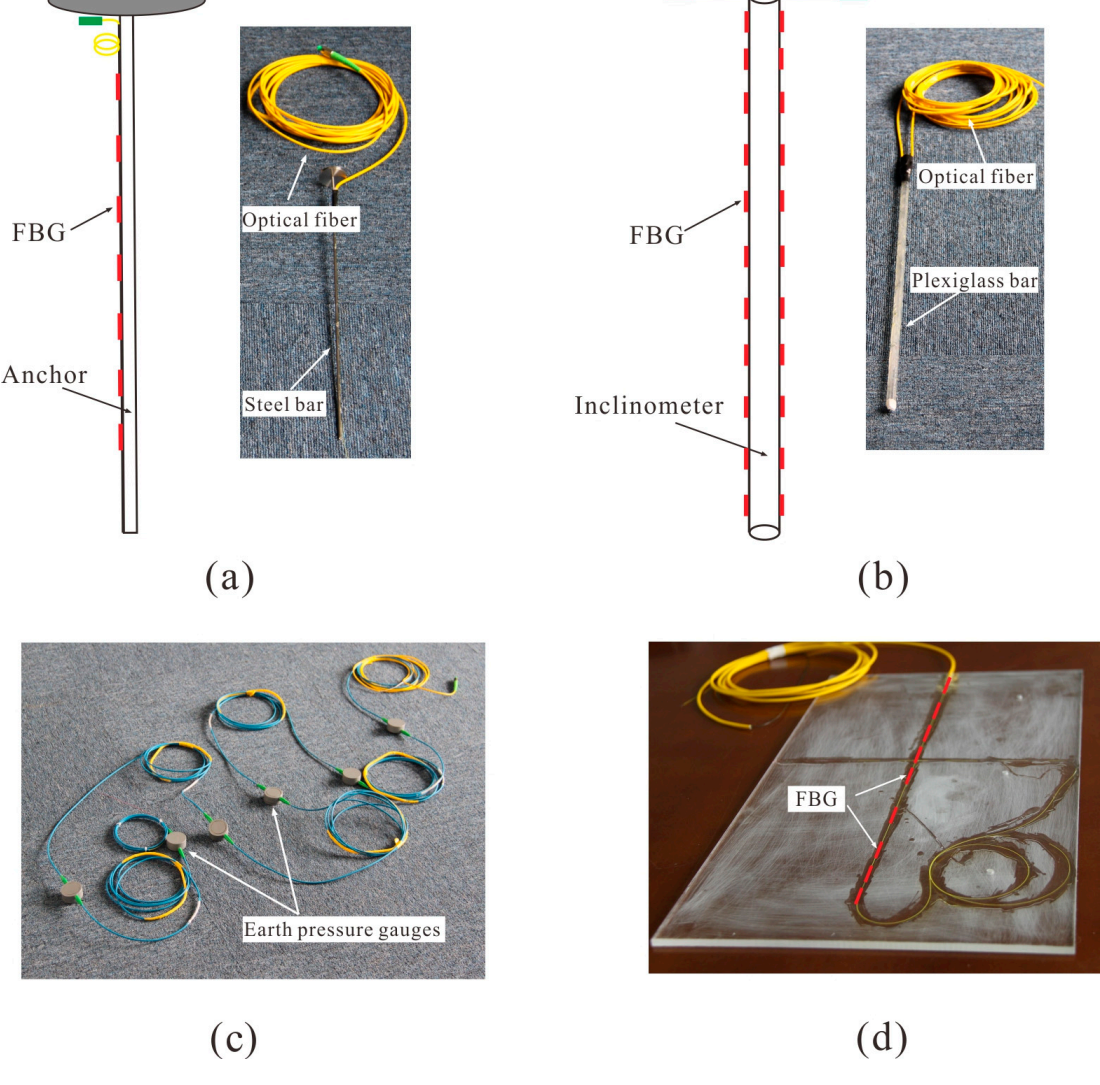
Developed sensors: (**a**) Anchors (**b**) Inclinometer pipe (**c**) Earth pressure gauges (**d**) Retaining wall.

The shear forces are gradually induced at the critical surface with the increase of centrifugal force, where the anchor mainly reinforces the slope by exerting tensile force [19]. That is, the tensile strength is the main test quantity, and the object with a similar Young’s modulus to the anchor prototype is usually selected in the model test. In this paper, two Φ 3 mm stainless steel bars with a steel disk at one end, whose length were 40 cm and 34 cm, were used to simulate the anchors (Figure 3a). The surface of the stainless steel bars is roughened with sandpaper to increase the bonding effect of glue. Then, both ends of the grating series (the grating series for the upper and lower anchors consist of seven bare gratings and 10 bare gratings, respectively) were fixed by quick-drying glue on one side of the anchors. Please note that a bare grating means the fiber jacket coating is stripped down to the cladding. Each bare grating is 10 mm in length. Then, the whole grating series was applied with curing adhesive and cured for 6 h at high temperature. Finally, a layer of epoxy resin was applied on the surface of the grating string and cured for 24 h. In order to minimize the influence of the inclinometer on the slope deformation, a tiny plexiglass tube (45 cm in length and 10 mm in diameter) was chosen as a substrate for the FBGs. The manufacturing process of the inclinometer pipe is similar to that of the anchors. Two grating strings, each consisting of 10 bare grating in series were glued symmetrically at the opposite sides of the inclinometer pipe, as shown in Figure 3b. It should be stated that due to the poor heat resistance of plexiglass, ultraviolet adhesive is selected as curing agent instead of epoxy resin.

#### 2.2.2. Earth Pressure Gauge and Retaining Wall

A plexiglass plate, with a height of 25 cm and a width of 6 cm, was used to simulate the retaining wall (Figure 3d). An FBG array, consisting of nine bare gratings, was glued along the central axis of the retaining wall. In order to meet the needs of centrifuge monitoring, the earth pressure gauges (Φ 40 × 16 mm) (Figure 3c), installed at the bottom of the model slope, have been designed. The pressure membrane attached with FBG is stuck to the sensitive plate of the earth pressure gauges. When the earth pressure changes, the Bragg wavelength of FBG changes with the deformation of the pressure membrane. The linear relationship between the Bragg wavelength and the earth pressure can be determined through the laboratory calibration tests. The FBG arrays deployed in the substrate are small and lightweight, therefore they do not affect the characteristics of the substrate. Different types of sensors composed by a substrate and FBGs can realize an FBG-based monitoring network.

## 3. Laboratory Calibration

Considering the error amplification of the centrifuge, the accuracy of monitoring must be guaranteed. In [29], Zhang et al. mounted a pair of FBGs and resistance strain gauges onto a steel bar, in order to assess the measurement accuracy under loading. After a detailed examination of the recorded data, we derived that the average relative error of the FBGs is 1.9%, which is much smaller than that of resistance strain gauges, with an error of 9.3%. Before centrifugal tests, the relation between the strain variation and the corresponding wavelength shift in the FBGs and the accuracy of the FBG-based sensors has been characterized through calibration tests.

### 3.1. Calibration of the FBGs

**Figure 4:**
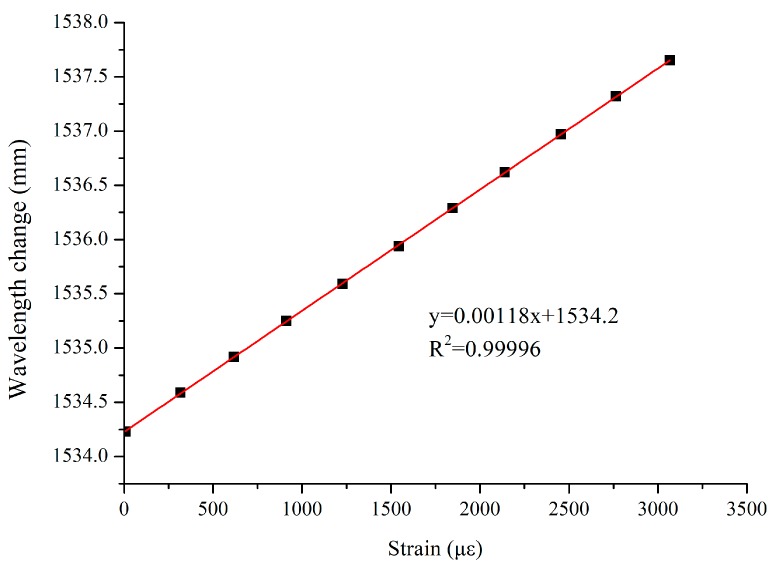
Calibration of bare gratings.

A tensile stand was used for the calibration of the bare gratings used in the centrifugal test. To this aim, the two ends of a 2 m-long fiber containing the gratings were fixed to the tensile testing apparatus. The test was carried out by applying progressively a stretching displacement, with an increment of 0.6 mm (300 με), the amount of stretching being controlled by a dial gauge. The results revealed a good linear relationship (Figure 4) between strain and FBG wavelength shift, with a correlation coefficient R^2^ equal to 0.99996.

### 3.2. Calibration of Earth Pressure Gauges and Anchors

**Figure 5:**
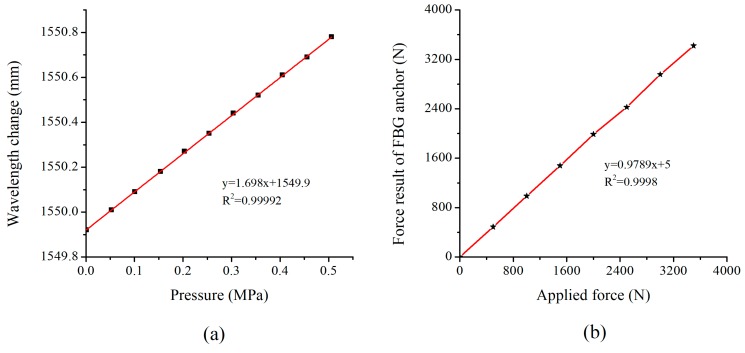
Calibration of FBG sensors (**a**) Calibration of earth pressure gauge (**b**) Calibration of FBG-based anchor.

A piston type hydraulic pressure regulator was used to calibrate the FBG-based earth pressure gauge. Twelve loading steps, ranging from 0 to 0.5 MPa, were applied to the earth pressure gauge. As shown in Figure 5a, a good linear relationship was found between the FBG wavelength shift and the pressure variation, with a correlation coefficient equal to 0.99992.

Similar to the bare grating calibration, the lower FBG-based anchor was placed in the tensile stand with one end fixed. A force ranging from 500 to 4000 N with an increment of 500 N was applied to the anchor. Each step was applied after reaching a stable condition in the previous step. After the calibration test, the strain values measured by 10 FBGs at each stage of the lower anchor were averaged, and the corresponding axial force was calculated according to Equation (3). The relationship between the applied force and the result of FBG anchor is shown in Figure 5b, revealing a good agreement between the values estimated through the FBG-based anchor and the actual values.

### 3.3. Calibration of Inclinometer Pipe

**Figure 6:**
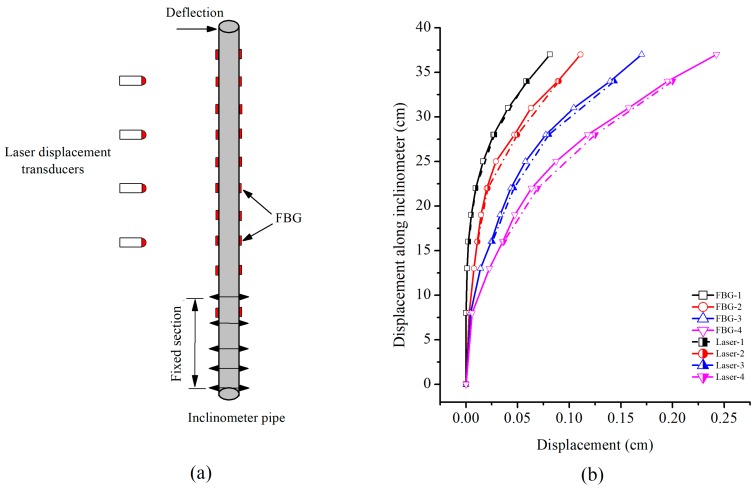
Calibration of the inclinometer pipe (**a**) Simple diagram of the calibration test (**b**) Comparison of the displacement obtained by the FBG-based inclinometer and laser displacement transducers.

The calibration of inclinometer pipe is illustrated in Figure 6. The diagram of the calibration test is shown in Figure 6a. Different deflection displacements were applied on top of the inclinometer pipe, which was kept fixed on its bottom for a 10 cm length. Four laser displacement transducers, located at a depth of 16, 22, 28 and 34 cm, were used to record the displacement during the tests. One thing that needs to be pointed out is that black ink was applied on one side of the inclinometer pipe against the laser displacement transducers to prevent the laser transmission in the plexiglass pipe. The displacements obtained by the FBG data, and those provided by the laser displacement transducers are compared in Figure 6b. The average relative error of each test is 1.25, 2.55, 4.67, and 4.80%, indicating a quite high measurement accuracy.

## 4. Centrifuge Model Test

**Figure 7:**
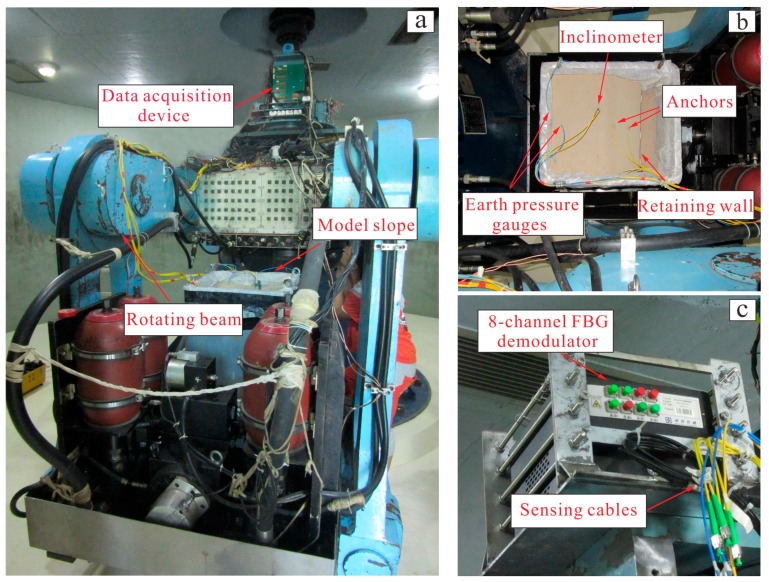
Centrifugal model test (**a**) Photograph of the centrifuge (**b**) Model slope installed on the centrifuge (**c**) Data acquisition device.

**Figure 8:**
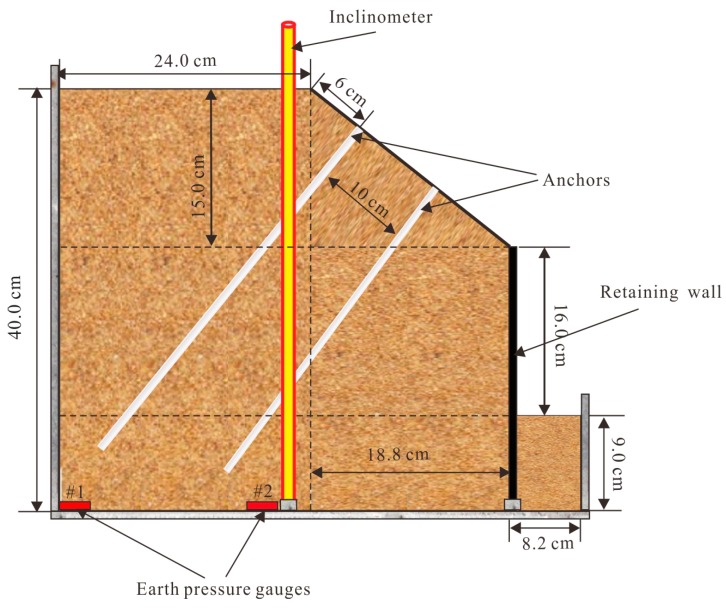
Schematic view of the centrifuge model.

In order to verify the reliability and applicability of the FBG monitoring system, a centrifugal model slope test was carried out in Tongji University in China. Figure 7 shows the diagrammatic sketch of the centrifugal test device. The device consists essentially in three parts: (1) the centrifuge (Figure 7a), through which the centrifugal force was applied on the model slope; (2) the model slope (Figure 7b), in which the FBG monitoring system was installed; (3) the data acquisition device (Figure 7c), through which the results of FBG-based sensors were recorded. The centrifugal force was increased by applying increasing angular velocity on the rotating beam, as shown in Figure 7a. The model dimensions were 51 × 45 × 55 cm (length × width × height), while the chamber walls were made of steel plates. Figure 8 provides a schematic view of the centrifuge model. The slope model was 40 cm high, including a slope height of 31.0 cm and a base height of 9.0 cm. It was constructed with an inclination of 1V:1.25H. The soil was a mixture of 80% sand and 20% kaolin by dry weight with an initial water content of 10%. The soil had a specific gravity of 2.52, and a unit weight of 19.8 kN/m^3^. The result of Proctor compaction tests showed that the optimum moisture content and maximum dry unit weight were 11.2% and 18 kN/m^3^, respectively. The reinforcement structures consisting of inclinometer pipe, anchors and retaining wall are shown in Figure 8.

The soil material was a blended silty clay. Due to the existence of voids between the particles, excessive settlement occurred during the centrifuge operation. Therefore, during the filling process, the layered compaction method was adopted to construct the model slope. The FBG-based sensors were embedded in the designed position, as shown in Figure 8. 

Specifically, the bottom of the inclinometer pipe and the retaining wall were fixed. At this stage, the working condition of the FBG-based sensors was detected to ensure the normal operation of the whole monitoring network. Before the centrifugal test, the slope model was kept static for 48 h in order to ensure the consistency of the soil mass and sufficient coupling between soil and sensors. Then, the model box was installed on the centrifuge, and the sensors were connected to the 8-channel FBG demodulator (Figure 7c) through the optical cables. During the test, the centrifuge was accelerated from 0 g to 60 g with an increment of 5 g, and the data of the sensors were recorded in real time. Until no obvious change in data, the centrifuge was accelerated to the next stage.

## 5. Test Result and Analysis

### 5.1. Anchors Test Result

**Figure 9:**
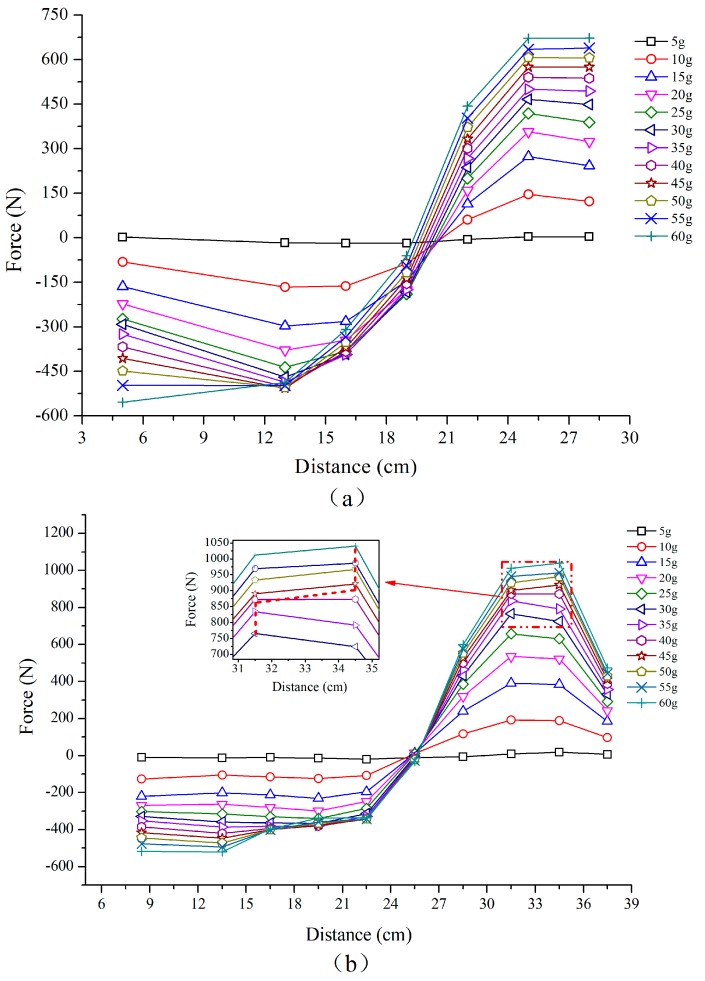
Axial force of anchors (**a**) The upper anchor (**b**) The lower anchor.

After conversion of the strains according to Equation (3), the axial forces of the anchors are illustrated in Figure 9. Each marker corresponds to an FBG. The distance here refers to that from the anchor toe. It can be found that the axial force in the upper and lower anchors exhibited similar variation characteristics. During the test process, the front part of anchor was in a compressed state, which can be attributed to the compression of soil due to the high centrifugal force. Contrarily, the rear part was subject to tension, indicating a gradual development of shearing of the soil mass (Figure 9a,b). 

In addition, the axial force exhibited a stepped increase with the increase of centrifugal force. When the centrifugal force is small, the axial force of upper and lower anchor is almost the same. When the centrifugal force increases, the axial force on the lower anchor exceeds that acting on the upper one. Actually, as the centrifugal force acting on the slope increases, the upper anchor cannot support enough retaining resistance to maintain the stability of the slope, and the lower anchor begins to play an important role in resisting the deformation of the slope. It is also important to note that the peak force of lower anchor moves to the rear part (from 31.5 cm to 34.5 cm) as the dash line shown in Figure 9b since the centrifugal force reached 45 g. When the slope instability is induced, the shear force accumulates at critical zones, and the peak tensile force of the anchors can therefore be used to locate the sliding surface [19]. It can be concluded that the critical sliding surface moves towards the upper part of the slope when the centrifugal force is increased up to 45 g.

### 5.2. Inclinometers Test Result

**Figure 10:**
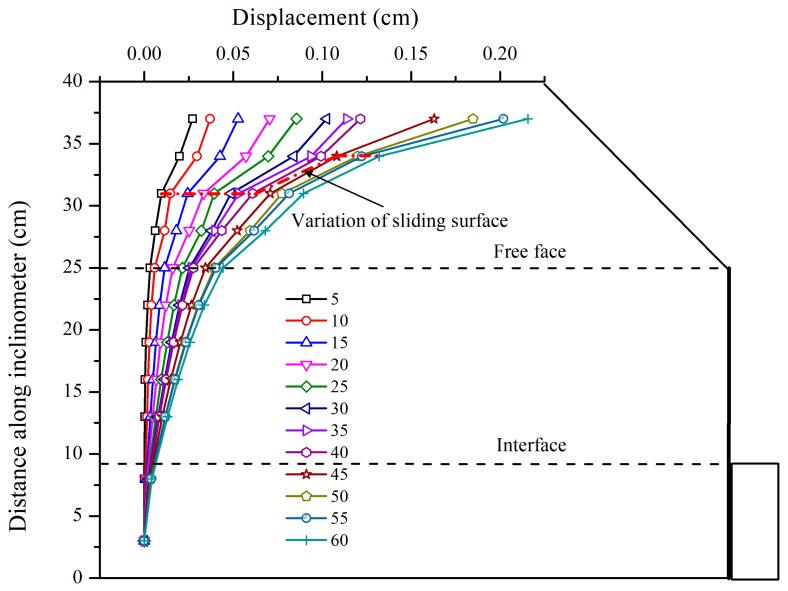
Displacement of inclinometer pipe for centrifugal forces ranging from 5 g to 60 g.

Figure 10 shows the horizontal displacement of FBG-based inclinometer pipe with respect to depth. It is found that the horizontal displacement increases with the increase of centrifugal force. Under the combined action of retaining wall and soil support, almost no deformation occurred from 9 cm down to the bottom of the inclinometer pipe. Due to the loss of the soil support, the inclinometer began to deform since it passed the interface as shown in Figure 10. However, the deformation of the inclinometer pipe is still at a low level, which means that the retaining wall played an obvious role in anti-sliding. The first jump displacement occurred at a depth of 25 cm, upon which the model lost the support of retaining wall. The maximum displacement, approximately 0.2 cm, occurred at the crest of the slope, which indicates the maximum deformation starting at the crest of the slope will involve the toe area gradually. Considering that the lower anchor exerts its resistance force later than the upper one, it can be concluded that the slope exhibits thrust load-caused deformation pattern. Zhang et al. has carried out similar test but without retaining wall, and found that the maximum displacement occurred near the slope toe initially, and then failure increased upwards [30]. Hence, it can be inferred that the retaining wall can somewhat change the deformation pattern of the slope. Increasing the centrifugal force from 5 g to 40 g, the potential sliding surface is detected at a the depth of 31 cm through a sharply increased displacement of the inclinometer pipe. However, as shown in Figure 10, the sliding surface moved to the depth of 34 cm when the centrifugal force was further increased from 45 g to 60 g.

### 5.3. Retaining Wall and Earth Pressure Test Result

**Figure 11:**
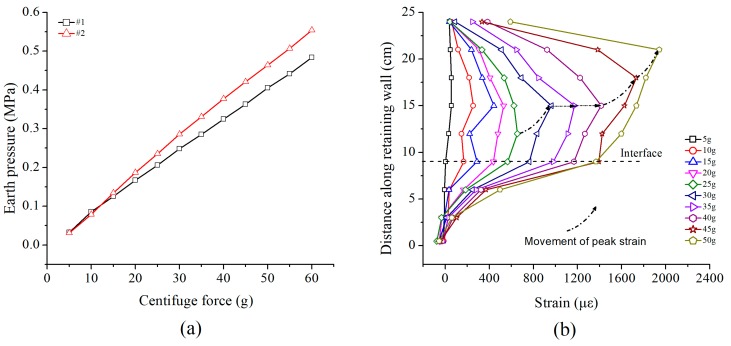
Test result of earth pressure and retaining wall. (**a**) Earth pressures (**b**) Strain curves of retaining walls.

The test results of the retaining wall and earth pressure gauges are illustrated in Figure 11. The earth pressure at the bottom increased linearly with the increase of centrifugal force as shown in Figure 11a. As the earth pressure gauge #1 was close to the boundary of the model slope, the friction between the soil and sidewall offset the weight of the soil to some extent, resulting in an earth pressure recorded by gauge #1 being about 0.07MPa smaller than gauge #2. 

The strain of the retaining wall (Figure 11b) showed an increasing tendency with increased centrifugal force. The maximum strain occurred in the middle area of the retaining wall, which is consistent with the result found by Li et al. [31]. When the centrifugal force exceeded 40 g, the strain increased significantly, which can be inferred as the retaining wall is exerting anti-sliding effect. The strain variation of the retaining wall cannot be collected when accelerated to 50 g. It can be inferred as the gratings were weakened due to the stripped of the fiber jacket at the beginning of gluing the grating series to the retaining wall (see Figure 3d). Consequently, the FBGs were broken while facing relative large centrifugal force. Similarly, the phenomenon of the movement of peak strain was also observed through the monitored data when the critical centrifugal force reached 45 g.

## 6. Discussion

The evolution of the model slope under an increasing centrifugal force was simulated with the numerical modeling software Fast Lagrangian Analysis of Continua (FLAC3D). The geometry of the simulated model was the same as the experimental model slope. Two vertical sides were assigned with a fixed displacement boundary condition in the x direction, while the bottom was fixed in both x and y direction. The model slope was assigned with the centrifugal force with an increment of 5 g. The numerical simulation adopted a Mohr-Coulomb failure criterion, and the parameters of the soil obtained by direct shear test, together with the sensors used in the FLAC3D analysis are listed in Table 1, Table 2 and Table 3.

**Figure 12:**
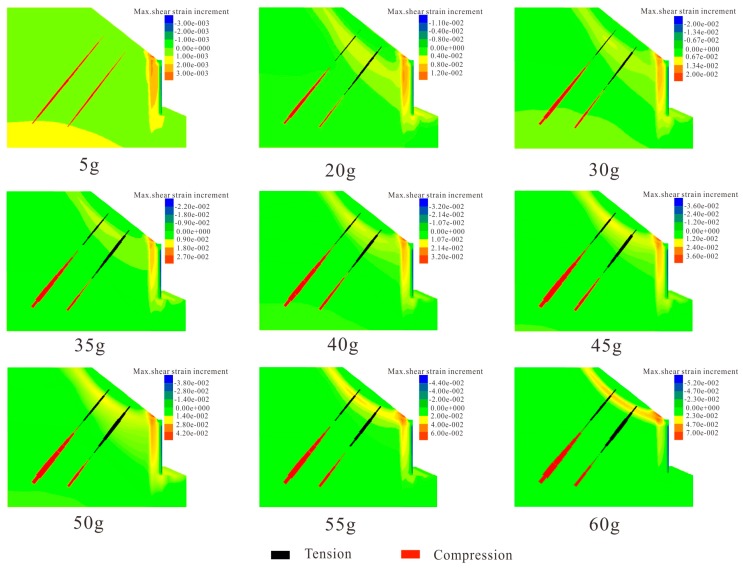
The simulated evaluation process of the model slope under different centrifugal force.

**Figure 13:**
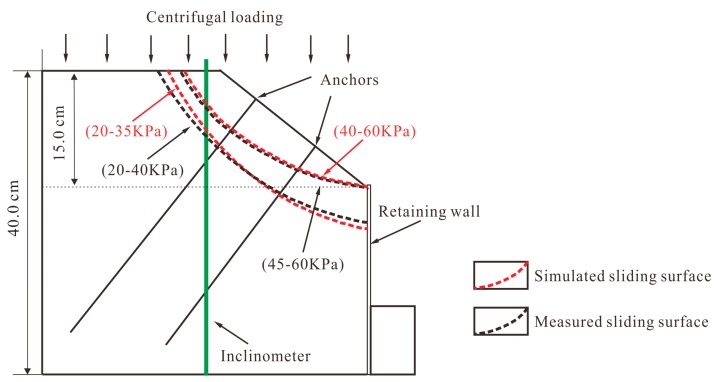
Slope failure mode under different centrifugal force.

The maximum shear strain increment contours in Figure 12 show the evolutionary process of the model slope under different centrifugal forces. When the applied centrifugal force was 5 g, the strain was mainly concentrated at the retaining wall, and the anchors were in a compressive state. With the increase of the centrifugal force, the shear strains propagated towards the middle area of the retaining wall and the critical sliding surface gradually formed. Due to the anti-sliding of the retaining wall, the sliding surface moved to the upper part of the slope at the critical centrifugal force of 40 g. According to the data provided by the FBG-based sensors, the failure mode of the model slope, under increased centrifugal force, are shown in Figure 13. The location of the simulated and measured sliding surfaces, represented by red and black dashed lines, are comparatively consistent. However, the critical centrifugal force for the transition of the sliding surface are different. The variation of the sliding surface obtained by measured method lagged 5 g than that of simulated method. This can be explained as follows: when the centrifuge rotates at a constant speed, the centrifugal force increases with the rotation radius. However, the crest and bottom accelerations of the model slope differ by ω^2^*h* [32] (ω is the angular velocity and h is the height of the model slope). As the length of rotating beam is 3 m and the height of the model slope is 0.4 m, when accelerated to 45 g, the top centrifugal force is actually 39 g. It accounts for the difference of critical centrifugal force for the transition of the sliding surface between the measured and simulation results. 

Considering the same material and deformation calculation theory for the inclinometer pipe and the retaining wall, the anchors and inclinometer pipe were selected and the comparison between measured and simulated values were made to prove the accuracy and feasibility of the FBG-based monitoring system.

**Figure 14:**
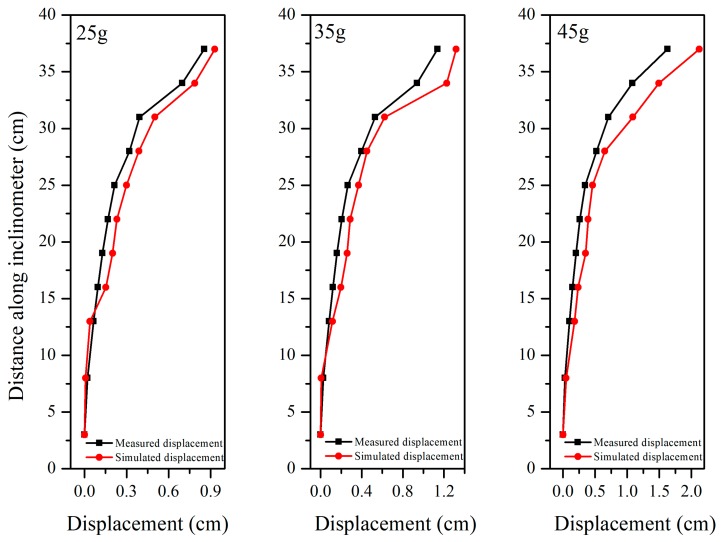
Comparison of the displacement obtained by the FBG-based inclinometer and simulation.

**Figure 15:**
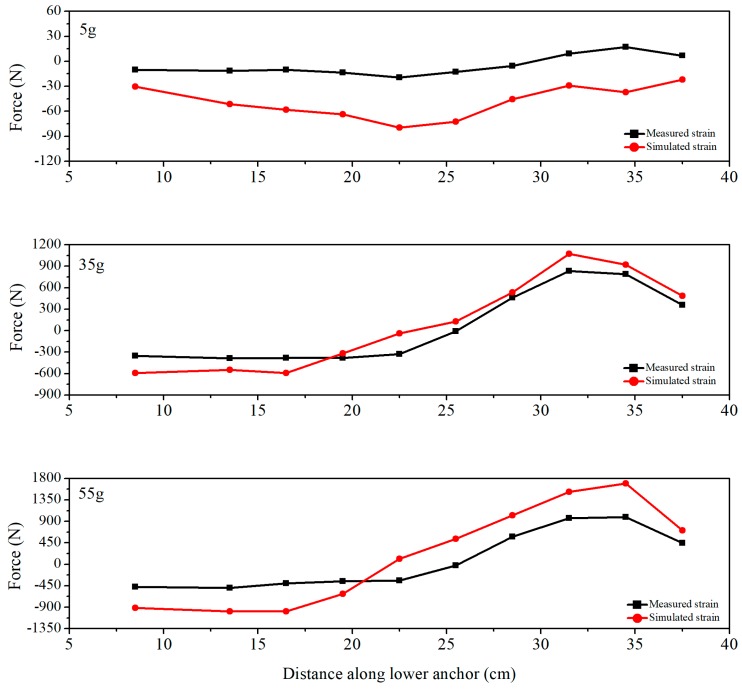
Comparison between the axial force provided by the FBG-based anchor and simulation.

It is noted that the internal displacement obtained by FBG-based inclinometer was essentially consistent with the numerical simulation under different centrifugal forces, as shown in Figure 14. However, because of the effect of friction and the inhomogeneity of soil around the inclinometer pipe due to compaction, the displacement obtained by simulation is generally larger than that of the measured result. A similar situation occurs when comparing the axial forces (Figure 15). The reasons mentioned above can also explain why the measured strain was smaller than the simulated strain at a centrifugal force of 5 g. After increasing the centrifugal force up to 35 g, the numerical and experimental results are in good agreement.

**Figure 16:**
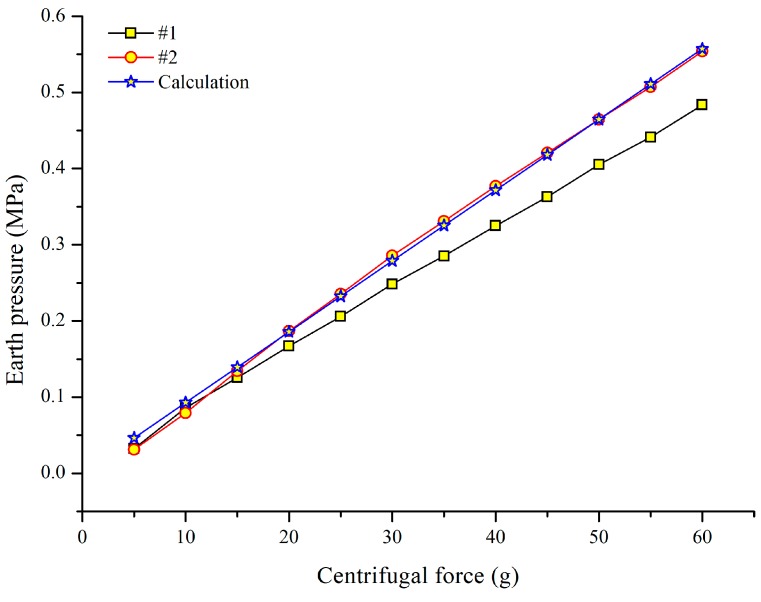
Comparison of the earth pressure obtained by FBG-based gauges and calculation.

However, increasing the force up to 55 g, the simulated strain was larger than the measured one. It can be inferred that, due to the smooth surface of the anchor, slippage between the anchor and the soil occurs under the action of a relatively large centrifugal force. Generally speaking, under the action of a certain centrifugal force, the anchor can work very well. Beyond that, based on the earth pressure calculation method, the measured results of earth pressure gauge #2 agreed well with calculations (Figure 16). 

Overall, the simulated and measured inclinometer displacements and anchor forces have close values and variation tendency. Besides, the measured earth pressure and calculated one agree very well. Therefore, the FBG-based monitoring system is feasible.

## 7. Conclusions

In the light of deficiencies of existing monitoring methods, an FBG-based monitoring system was introduced and applied in a geotechnical centrifuge. Its validity has been demonstrated by comparing the results of FBG-based sensors and those of a numerical simulation. The key findings and conclusions are summarized below:

(1). An FBG-based monitoring system was developed. Compared to conventional sensors, the proposed system has the unique advantages of low weight, small volume, immunity to electromagnetic interference and quasi-distributed sensing, which makes it especially attractive for centrifugal tests.
(2). According to the laboratory calibration test, the FBG monitoring system can monitor the multi-parameter information of the slope, including the stress (the earth pressure and anchor force), the displacement (inclinometer pipe deformation) and strain (retaining wall bending) of the model slope with high accuracy.
(3). The stress state of the anchors, the strain distribution of the retaining wall together with the displacement of the inclinometer pipe depict the failure mode of the model slope, and change when increasing the centrifugal force. Furthermore, we have found that the critical centrifugal force for the transition of the sliding surface is 45 g.
(4). The maximum displacement occurred at the crest of the slope, therefore the maximum deformation starting at the crest of the slope will reach the toe area gradually. Considering that the lower anchor exerts its resistance force later than the upper one, we conclude that the slope exhibits thrust load-caused deformation pattern.
(5). Based on the developed FBG monitoring system, multi-parameter information of centrifugal slope can be obtained and further comprehensive analysis on evolution mechanism and stability evaluation can be carried out.

## Figures and Tables

**Table 1:** Properties of the soil in the model test.

Unit Weight *Γ* (kN/m^3^)	Elastic Modulus *E* (MPa)	Poisson’s Ratio	Friction Angle *Φ* (°)	Cohesion *C* (kPa)
19.8	58	0.3	25.4	5.2

**Table 2:** Properties of the inclinometer and retaining wall.

Material	Bulk Modulus *K* (MPa)	Shear Modulus *G* (MPa)
Plexiglass	870	430

**Table 3:** Properties of the anchor.

Material	Elastic Modulus *E* (GPa)	Tensile Strength (MPa)
Steel	95	600
